# Molecular characterization of potential *Plasmodium*-Blocking *Serratia* spp*.* bacteria in field-caught malaria mosquito in Burkina Faso

**DOI:** 10.1186/s13071-025-07191-2

**Published:** 2025-12-21

**Authors:** Haoua Traoré, Edounou Jacques Gnambani, Domonbabele François de Sales Hien, Raymond Karlhis Yao, Maurice Konkobo, Aicha Fatimata Sodré, Martin Bienvenu Somda, Abdoul Salam Ouedraogo, Abdoulaye Diabaté, Etienne Bilgo

**Affiliations:** 1 Laboratoire de Recherche Sur Les Maladies Infectieuses Et Parasitaires (LR-MIP), Institut de Recherche en Sciences de La Sante (IRSS), Centre National de La Recherche Scientifique Et Technologique (CNRST, Bobo-Dioulasso, Burkina Faso; 2 Centre Muraz, Institut National de Santé Publique (INSP), Bobo Dioulasso, Burkina Faso; 3 Centre d’Excellence Africain en Innovations Biotechnologiques Pour L’Elimination Des Maladies À Transmission Vectorielle (CEA/ITECH-MTV), Université Nazi Boni (UNB), Bobo-Dioulasso, Burkina Faso; 4 Centre International de Recherche-Développement Sur L’Elevage en Zone Subhumide (CIRDES), Bobo-Dioulasso, Burkina Faso; 5 Laboratoire de Bactériologie Et de Virologie, Centre Hospitalier Universitaire Souro Sanou, Bobo Dioulasso, Burkina Faso; 6 Laboratoire Des Pathogènes Émergents Et Réémergents, Ecole Doctorale Sciences de La Santé, Université Nazi BONI, Bobo Dioulasso, Burkina Faso

**Keywords:** Malaria, *Serratia*, *Plasmodium*, Mosquitoes, Phylogenetic, West Africa

## Abstract

**Background:**

One of the alternatives for controlling malaria is using mosquito symbiotic bacteria to reduce *Plasmodium* transmission. Species of *Serratia,* a genus of the Enterobacteriaceae family, have been isolated from the midgut of mosquitoes and are commonly found in water, soil and plant surfaces. These bacteria have shown great promise in blocking the transmission of *Plasmodium* in mosquitoes. The aim of this study was to isolate and characterize the genus *Serratia* within the *Anopheles gambiae* complex from Burkina Faso.

**Methods:**

Mosquitoes were collected in three field sites located in Houet Province in western Burkina Faso (Dioulassoba, Vallée du Kou and Soumousso), transported to the laboratory and identified morphologically. The salivary gland, midgut, spermatheca, ovary of females and testis of males were dissected and their contents ground up. Different species of *Serratia* were identified by PCR targeting of the* luxS* gene of *Serratia,* followed by 16S ribosomal RNA (rRNA) sequencing.

**Results:**

Molecular analyses identified the isolates as belonging to the genus* Serratia*, and phylogenetic reconstruction revealed that these strains are highly similar to one another but distinct from* Serratia* strains previously reported in neighboring countries such as Ghana and Nigeria. The overall prevalence of *Serratia* among malaria vectors was 13.3%. This prevalence varied according to the development stage of mosquitoes, locality of origin and mosquito organ. Only one *Anopheles coluzzii* mosquito was co-infected with *Serratia* and *Plasmodium falciparum*.

**Conclusions:**

The results of this study support the presence of *Serratia* spp. in wild mosquitoes from Burkina Faso, we well as their potential use in malaria control.

**Graphical Abstract:**

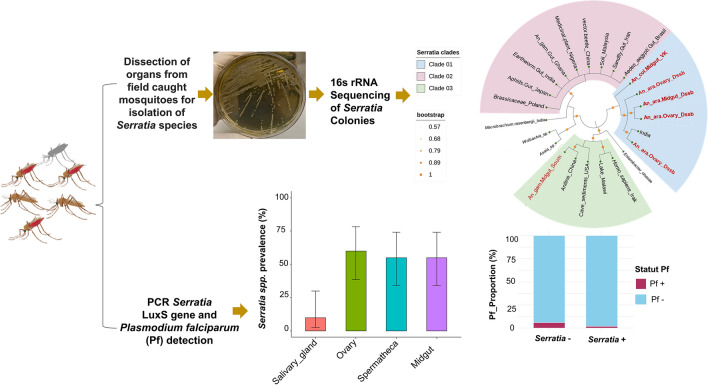

**Supplementary Information:**

The online version contains supplementary material available at 10.1186/s13071-025-07191-2.

## Background

The fight against malaria continues to encounter major obstacles, including climate change, humanitarian crises, resource constraints and biological challenges such as antimalarial drug resistance and insecticide resistance, even in the context of concerted global efforts [1, 2]. These issues significantly hinder advancement toward the goal of a malaria-free world. In 2024, the WHO reported a concerning rise to 263 million cases of malaria globally, marking an increase of 11 million cases since 2022 [3]. This increase underscores the critical need for innovative and complementary strategies to effectively fight this disease.

In this landscape, the exploration of non-pathogenic bacterial species as biological agents offers a promising avenue [4, 5]. Such bacteria can play a pivotal role in the malaria control arsenal, complementing existing tools and environmentally friendly and sustainable strategies [6]. The integration of bacterial agents into biological control programs is recognized as an emerging field with significant potential. In this context, the genus *Serratia*, part of the Enterobacteriaceae family in the Proteobacteria subclass, has garnered attention for its symbiotic relationships with various insect species [7–10]. For example, the *Serratia ureilytica* YN1 strain, isolated from the gut of *Anopheles sinensis* mosquitoes in Yunnan, China, a region declared malaria-free by the WHO [11], has demonstrated the capacity to inhibit the development of the *Plasmodium falciparum* parasite within its mosquito host via the action of AmLip, a lipase produced by this strain [12]. Also, the natural red pigment prodigiosin from a *Serratia marcescens* strain has shown the ability to inhibit *P. falciparum* under in vitro conditions [13], and the bio-products from another strain of *Serratia marcescens* isolated from Ghanaian *Anopheles gambiae* reduced *P. falciparum* development in mosquitoes [14]. In addition, *S. marcescens* secretes proteases and chitinases with larvicidal activity against *Anopheles dirus*, a malaria vector in Asia [15].

Given this background, we focused on the identification and characterization of indigenous *Serratia* strains present in the *An. gambiae* complex of western Burkina Faso and their potential role in malaria vector control.

## Methods

### Sample collection

**Fig. 1:**
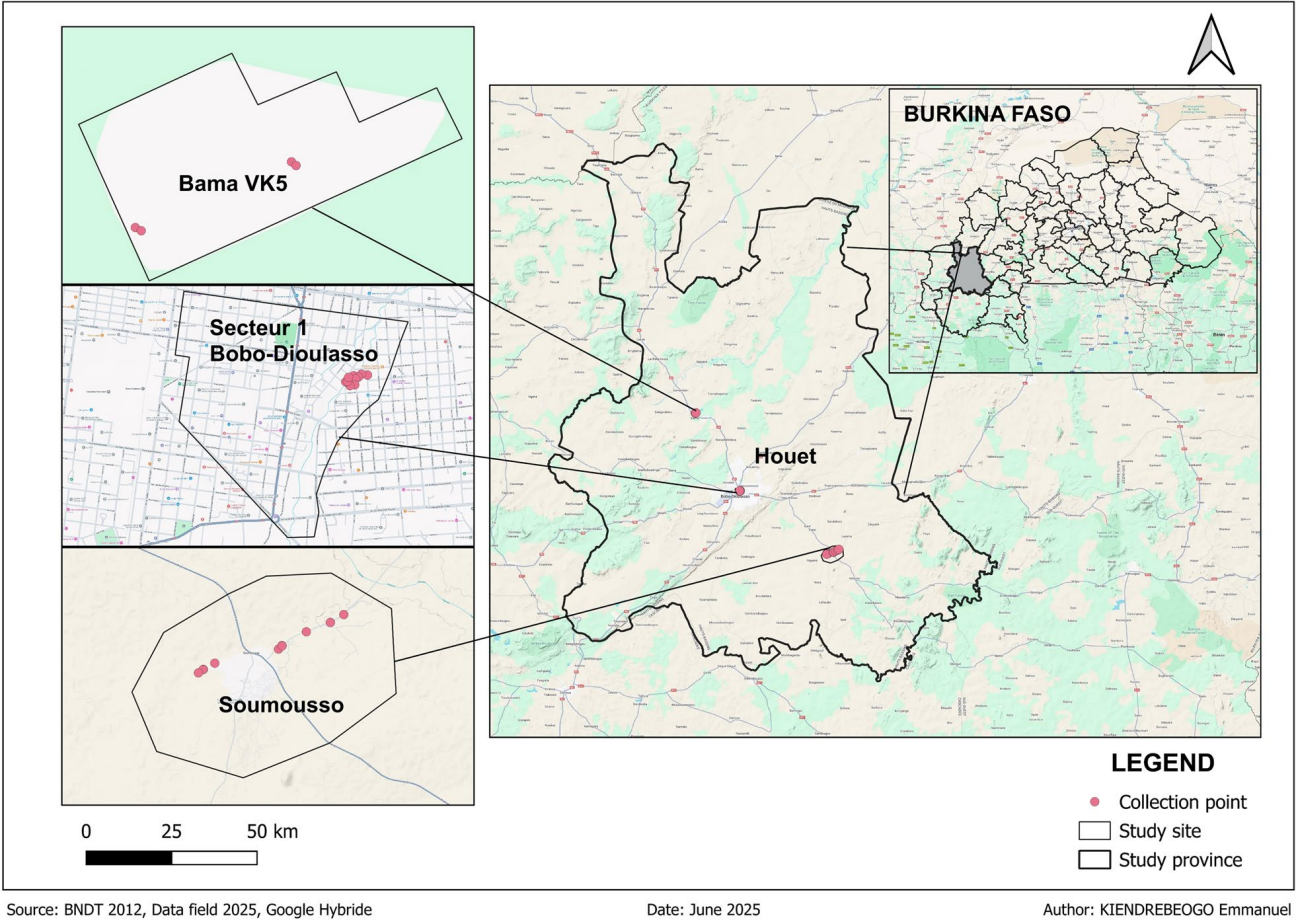
Map of mosquito collection area

Mosquitoes were collected in November 2024 in three districts (Dioulassoba [*N* = 275], Soumousso [*N* = 253] and Vallée du Kou [*N* = 254]) of Houet Province (western Burkina Faso) (Fig. 1).

Adult mosquitoes were sampled using the residual fauna collection method on all potential resting sites with aspirators, following WHO entomological guidelines [16]. However, larvae were collected from stagnant rainwater, brick pits and rice fields using the “deeping” method described in the manual on malaria entomology and vector control [16]. The larvae and adult mosquitoes were then transported to the laboratories of the Centre Muraz and the Institut de Recherche en Sciences de la Santé (IRSS) for molecular analysis.

### Determination of mosquito sex and feeding status

The collected mosquitoes were morphologically identified under a binocular microscope (Leica Microsystems, Wetzlar, Germany) as per the criteria established by Gillies and Coetzee [17]. Only mosquitoes from the *An. gambiae* complex were utilized for this study. Following morphological identification, the mosquitoes were counted based on their sex (male, female) and gonotrophic status (unfed, blood-fed and gravid).

### Dissection of mosquitoes, isolation and cultivation of microorganisms

Prior to dissection, mosquitoes were cold-anesthetized for 3–5 min at − 20 °C, and their cuticles were sterilized by soaking in 70% ethanol in 1.5-ml microtubes with shaking for 5 min. All dissection procedures were conducted in a Class II biological safety cabinet [18, 19]. The salivary glands, midgut, ovaries and spermatheca were dissected from each female mosquito, while only the testes and carcass were removed from male mosquitoes. Sets of five similar organs (e.g. 5 salivary glands, 5 ovaries, etc.) were pooled in 1.5-ml microtubes containing 1 ml of physiological saline according to the method of Kerri Coon (see Additional file 1: Text S1). Then, 100-µl aliquots of each crushed salivary gland, midgut, ovary, spermatheca and testis pool were inoculated onto a chromogenic medium plate by spreading uniformly over the surface of the medium and incubated at 37 °C for 24–48 h. The colonies were recovered according to the color of the dyes on the chromogenic detection medium. To isolate the pure bacterial colonies, we chose colonies of distinct size, shape and color for subculturing in Luria Broth (LB) at 37 °C for 24–48 h, following which the colonies were kept in 1-ml tubes containing LB and kept at 4 °C for further molecular analysis.

### Genomic DNA extractions

DNA from whole mosquitoes and from different organs of the mosquitoes was extracted using 2% cetyl trimethyl ammonium bromide (CTAB) (Additional file 1: Text S2).

### Molecular identification of mosquito species

The *An. gambiae* complex was molecularly identified using PCR methodology in which the anopheline species was identified by targeting insertion polymorphisms of *SINE200* [20], a small interspersed repeat element, using the primer pair S200X 6.1F (TCG-CCT-TAG-ACC-TTG-CGT-TA) and S200X 6.1R (CGC-TTC-AAG-AAT-TCG-AGA-TAC). Briefly, each sample was placed in a total reaction volume of 20 µl (2 µl DNA, 4 µl Master Mix [5× FIREPol Master; Solis BioDyne, Tartu, Estonia ], 0.4 µl of 10 μM primers for each primer and 13.2 µl ultrapure water). The PCR cycling conditions consisted of an initial denaturation at 95 °C for 15 min; followed by 35 cycles of denaturation at 95 °C for 30 s, annealing at 58 °C for 45 s and extension at 72 °C for 45 s; with a final elongation at 72 °C for 5 min. PCR products were visualized by electrophoresis in a 2% agarose gel, with expected fragment sizes of 479 bp for *Anopheles* *coluzzii*, 249 bp for *An. gambiae,* and 223 bp for *Anopheles arabiensis* (Additional file 2: Figure S1a).

### Molecular identification of *P. falciparum*

A total of 324 mosquitoes were screened for *P. falciparum* infection by conventional PCR using two specific primers: *P.falciparum*1 (5′-GGAATGTTATTGCTAACAC-3′) and *P.falciparum*2 (5′-AATGAAGAGCTGTGTATC-3′) [21]. The final total PCR reaction volume for one sample was 20 µl (2 µl DNA, 4 µl Master Mix (5×  FIREPol Master [Solis BioDyne]), 0.4 µl of 10 μM primers for each primer and 13.2 µl ultrapure water). PCR cycling was performed in a Flexlid Mastercycler Nexus Thermal Cycler (Eppendof AG, Hamburg, Germany), and the cycling conditions were: an initial cycle of 94 °C for 3 min; followed by 32 cycles of 94 °C for 30 s, 56 °C for 1 min 15 s and 68 °C for 1 min; with a final elongation at 68 °C for 5 min. Visualization of fragments at the expected size of 450 bp indicated the sample was positive for *P. falciparum* (Additional file 2: Figure S1b).

### Molecular identification of the *Serratia* genus

The 27F (AGAGTTTGATCCTGGCTCAG) and 1492R (TACGGYTACCTTGTTACGACTT) primer pair, which amplifies the 16S ribosomal RNA (rRNA) gene, was used to confirm the presence of bacterial DNA [22]. *Serratia* spp*.* identification was confirmed by PCR targeting of the specific* luxS* gene involved in quorum signal detection [23] using the primer sequences FluxS1 (GCTGGAACACCTGTTCGC) and RluxS2 (ATGTAGAAACCGGTGCGG). Quorum sensing is the ability of bacteria to communicate and coordinate behavior by emitting signaling molecules. The same reaction mix was used for amplification of 16S rRNA and the* luxS* gene: 13 µl of ultrapure water, 4 µl FIREPol Master Mix (5× FIREPol Master [Solis BioDyne], 0.5 µl of each primer and 2 µl of DNA). Amplification was carried out according to the following program: an initial cycle of 95 °C for 5 min; followed by 30 cycles at 95 °C for 15 s, 60 °C for 15 s and 72 °C for 30 s; with a final extension at 72 °C for 5 min; the amplicons were maintained at 10 °C. PCR products were visualized by electrophoresis in a 2% agarose gel, with expected fragment sizes of 102 bp for *Serratia* spp. and 1400–1500 bp for the 16S rRNA gene (Additional file 3: Figure S1).

### Sequencing of 16S RNA gene

The PCR amplicons of the 16S rRNA gene were sequenced by a commercial biotechnology company (GenoScreen, Lille, France) using Sanger sequencing technology—Formula One Shot. Sequences were further trimmed with FinchTV (Geospiza, Inc., Denver, CO, USA) and analyzed for confirmation using nucleotide BLAST (http://blast.ncbi.nlm.nih.gov/Blast.cgi) to compare* Serratia* taxa (taxon ID: 613) in the National Center for Biotechnology Information (NCBI) taxonomy database; other bacterial sequences served as outgroups for the phylogenetic trees (Additional file 4: Table S1).

### Phylogenetic analysis

The identified sequences were aligned with each available sequence’s representative of *Serratia* species and other bacteria (Additional file 5), using the Muscle program in MEGA 12.0 software (Additional file 6: Text S1). The phylogeny was inferred using the maximum likelihood method and Kimura two-parameter model with the invariant sites model in nucleotide substitutions, which is the best model generated with MEGA 12.0 [24]. The statistical robustness of the clusters was evaluated by bootstrap analysis after 1000 replications. The phylogenetic tree has been edited with iTOL v7.

### Statistical analysis

The data were analyzed using R software (version 4.0.3; R Foundation for Statistical Computing, Vienna, Austria). The generalized linear model (GLM) with a quasi-binomial distribution was used to determine the interaction effect of mosquito species and location of origin on *Serratia* infection status in mosquitoes. A McNemar’s chi-square test was used to compare the proportions of *Serratia* according to mosquito organs or female status (gravid, fed, unfed) (Additional file 7. Text S1). *P* < 0.05 was considered to indicate statistical significance.

## Results

PCR targeting of of the* luxS* gene of *Serratia* was performed on 782 mosquitoes of the *An. gambiae* complex, including 177 larvae and 605 adults from Soumousso (Soum), Dioulassoba (Dssb) and Vallée du Kou (VK). The overall prevalence of *Serratia* spp*.* was 13.3% (*n* = 104). The prevalence of *Serratia* spp. was significantly higher in adult mosquitoes (11.4%; *n* = 89) than in larvae (1.9%; *n* = 15) (*χ*^2^_(1)_ = 4.62, *P* = 0.03) (Table 1). Also, the prevalence of *Serratia* was significantly higher in female mosquitoes (12.4%; *n* = 75) than in their male counterparts (2.3%; *n* = 14) (*χ*^2^_(1)_ = 6.66, *P* = 0.01) (Table 2).

**Table 1:** Proportion of *Serratia* spp. in mosquitoes at different mosquito developmental stages

Stage	*Serratia*+	*Serratia*−
Larva	1.9% (15 /782)	66.0% (516/782)
Adult	11.4% (89/782)	20.7% (162 /782)

**Table 2:** Proportion of *Serratia* spp*.* in mosquitoes according to sex

Sex	*Serratia*+	*Serratia*−
Male	2.3% (14/605)	66.7% (367/605)
Female	12.4% (75/605)	24.6% (143 /605)

### Prevalence of *Serratia* in mosquitoes according to mosquito female status and organs

**Fig. 2:**
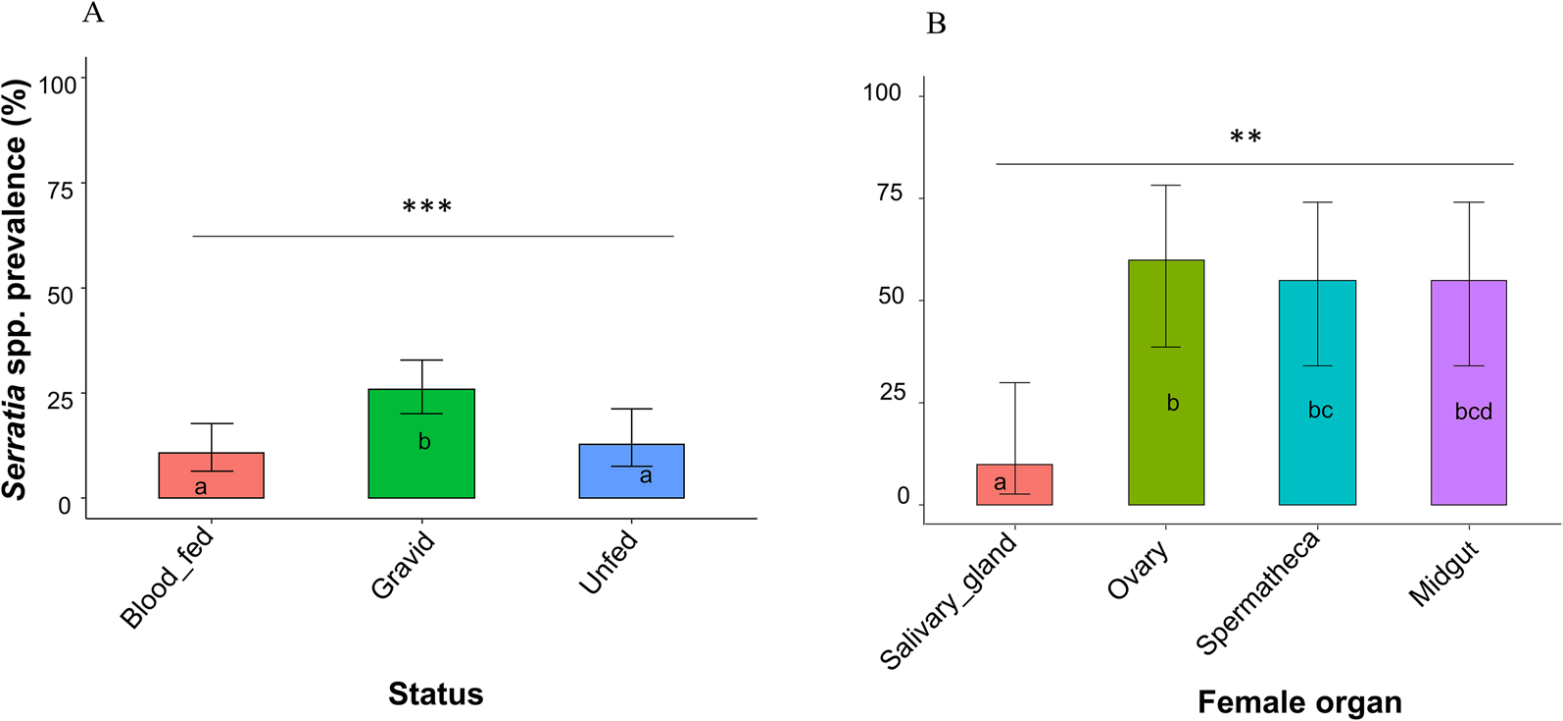
Prevalence of *Serratia* according to the status of female mosquitoes (Jeun [unfed], fed, gravid) (**A**) and specific organ (salivary gland, ovary, spermatheca, midgut) (**B**). Different lowercase letters indicate significant differences between groups at *P* < 0.05, with asterisks indicating the level of significance: **P < 0.01; **P < 0.001

The status (unfed, fed, gravid) of female mosquitoes significantly influenced the presence of *Serratia* (*χ*^2^_(2)_ = 133.4, *P* = 0.001). The proportion of *Serratia* was 26% (*n* = 47; 95% confidence interval [CI] 20–33) in gravid females, 11% (*n* = 13; 95% CI 6–18) in blood-fed females and 13% (*n* = 12; 95% CI 8–21) in unfed females (Fig. 2a). The proportion of *Serratia* also varied depending on the specific organ of female *Anopheles* analyzed (*χ*^2^_(3)_ = 13.33, *p* = 0.004). The highest prevalence of *Serratia* was observed in the ovaries (60%; *n* = 12; 95% CI 38–78), followed by the midgut and spermatheca (both 55%; *n* = 11; 95% CI 34–74), while the salivary glands showed the lowest prevalence of *Serratia* (10%; *n* = 2; 95% CI 3–30) (Fig. 2b). In organs of male mosquitoes, the prevalence of*Serratia* was 33.33% (*n* = 3; 95% CI 45–93) in the testes and 77.78% (*n* = 7; 95% CI 12–64) in the carcass; there was no statistically significant difference in *Serratia* prevalence between these latter two male organs (*χ*^2^_(1)_  = 5.3, *P* = 0.15).

### *Serratia* prevalence according to locality and *Anopheles* species

**Fig. 3:**
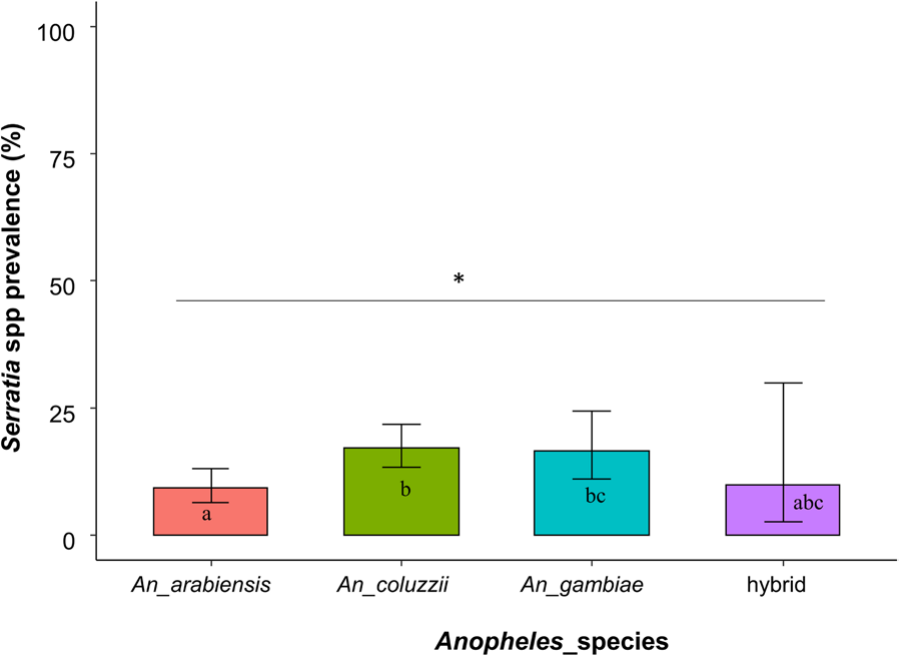
Prevalence of *Serratia* in three* Anopheles* species and hybrid according to the *Anopheles* species. Different lowercase letters indicate significant differences between groups at *P < 0.05

The overall proportion of *An. arabiensis* mosquitoes that tested positive for *Serratia* was 9.34% (*n* = 27; 95% CI 7–13); for *An. coluzzii* and *An. gambiae*, the proportion was 17.35% (*n* = 55; 95% CI 14–22) and 16.67% (*n* = 20; 95% CI 11–24), respectively (Fig. 3). The results also showed that* Anopheles* species and locality had an interaction effect on the infection status of *Serratia* spp*.* (*χ*^2^_(6)_ = 488.89, *P* = 0.02), with *Serratia* infection being statistically different depending on *Anopheles* species (*χ*^2^_(6)_ = 370.83, *P* = 0.03) and on locality (*χ*^2^_(5)_ = 371.64, *P* = 0.01).

**Fig. 4:**
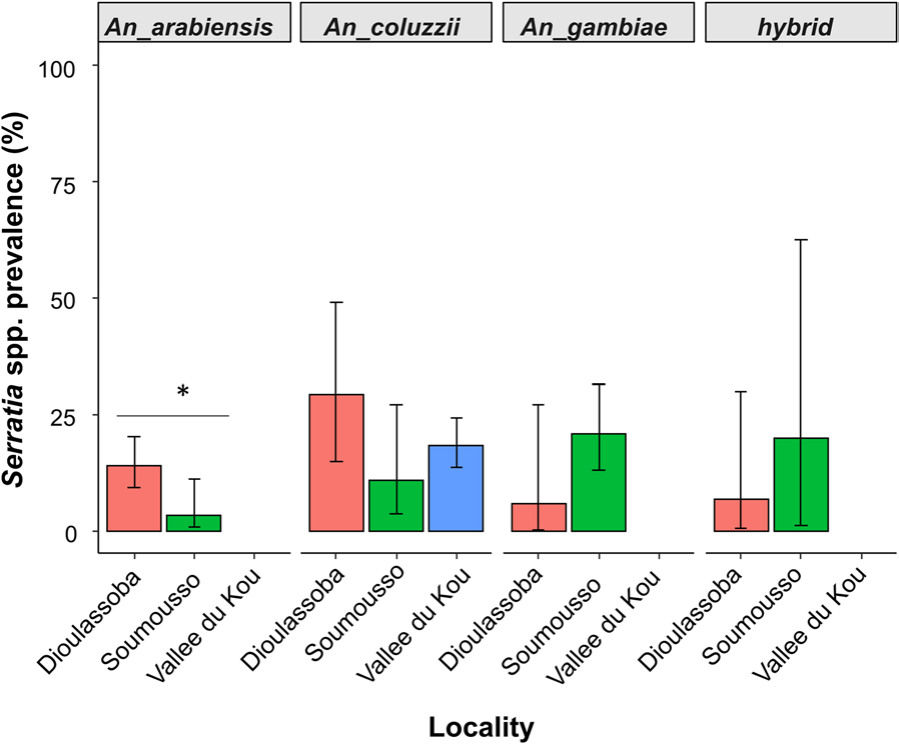
Prevalence of *Serratia* in three* Anopheles* species and hybrid according to the locality and *Anopheles* species. Asterisk indicates a significant difference at *P < 0.05

Regarding locality, the proportion of *An. arabiensis* mosquitoes that tested positive for *Serratia* was 13% (*n* = 22; 95% CI 9–20), 3% (*n* = 2; 95% CI 1–11) and 0% for Dioulassoba, Soumousso and Vallée du Kou, respectively (Fig. 4). The proportion of *An. coluzzii* mosquitoes that tested positive for *Serratia* was 29% (*n* = 7; 95% CI 14–49) for Dioulassoba, 11% (*n* = 3; 95% CI 4–27) for Soumousso and 18% (*n* = 37; 95% CI 14–24) for Vallée du Kou (Fig. 4). For *An. gambiae* species, *Serratia* was detected in 6% (*n* = 1; 95% CI 0–27), 21% (*n* = 15; 95% CI 13–32) and 0% for mosquitoes collected from Dioulassoba, Soumousso and Vallée du Kou, respectively (Fig. 4). Regarding hybrids, 7% (*n* = 1; 95% CI 0–30), 2% (*n* = 1; 95% CI 1–62) and 0% of mosquitoes collected from Dioulassoba, Soumousso and Vallée du Kou, respectively, tested positive for *Serratia* (Fig. 4).

### Impact of* Serratia* on* P. falciparum* in field-caught malaria mosquito vectors

**Fig. 5:**
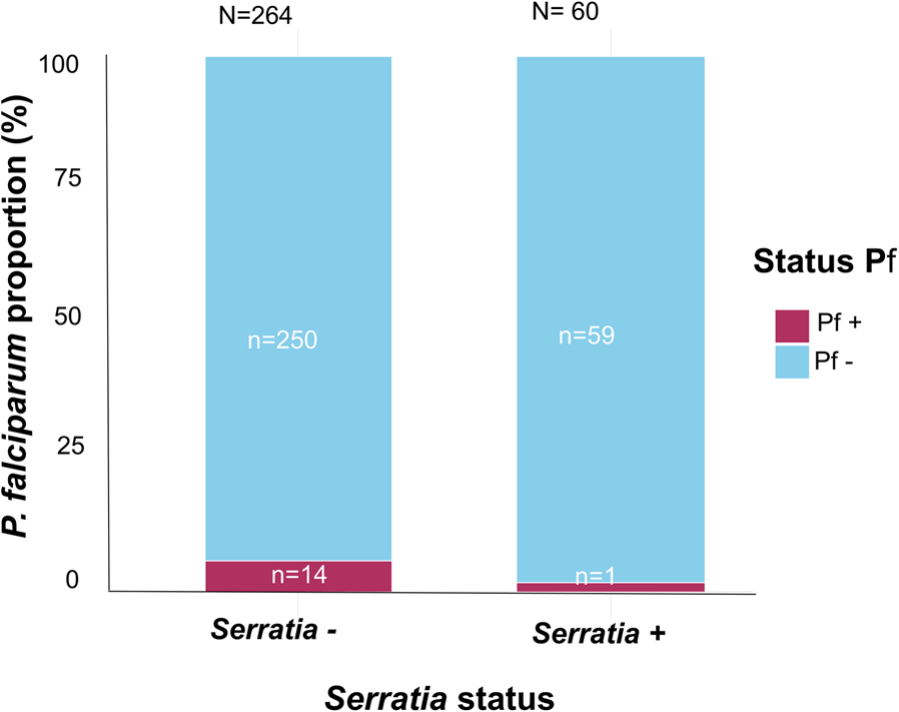
Prevalence of *Plasmodium falciparum* infection in wild-caught mosquitoes of the*Anopheles gambiae* complex. Pf, *Plasmodium falciparum*

Of the 324 field-caught *Anopheles* mosquitoes screened for *P. falciparum* infection by conventional PCR, the overall prevalence of *P. falciparum* infection was 4.6% (15/324). Regarding the prevalence of *P. falciparum* and *Serratia* co-infection in these mosquitoes, the results showed that 5.8% (*n* = 14) tested negative for *Serratia* and positive for *P. falciparum*, and that 1.7% (*n* = 1) of the mosquitoes tested positive for both *Serratia* and *P. falciparum* (Fig. 5). There was no significant association between the presence of *P. falciparum* and *Serratia* (*χ*^2^_(1)_ = 0.76, *P* = 0.4).

### Phylogenetic analysis of *Serratia* strains

**Fig. 6:**
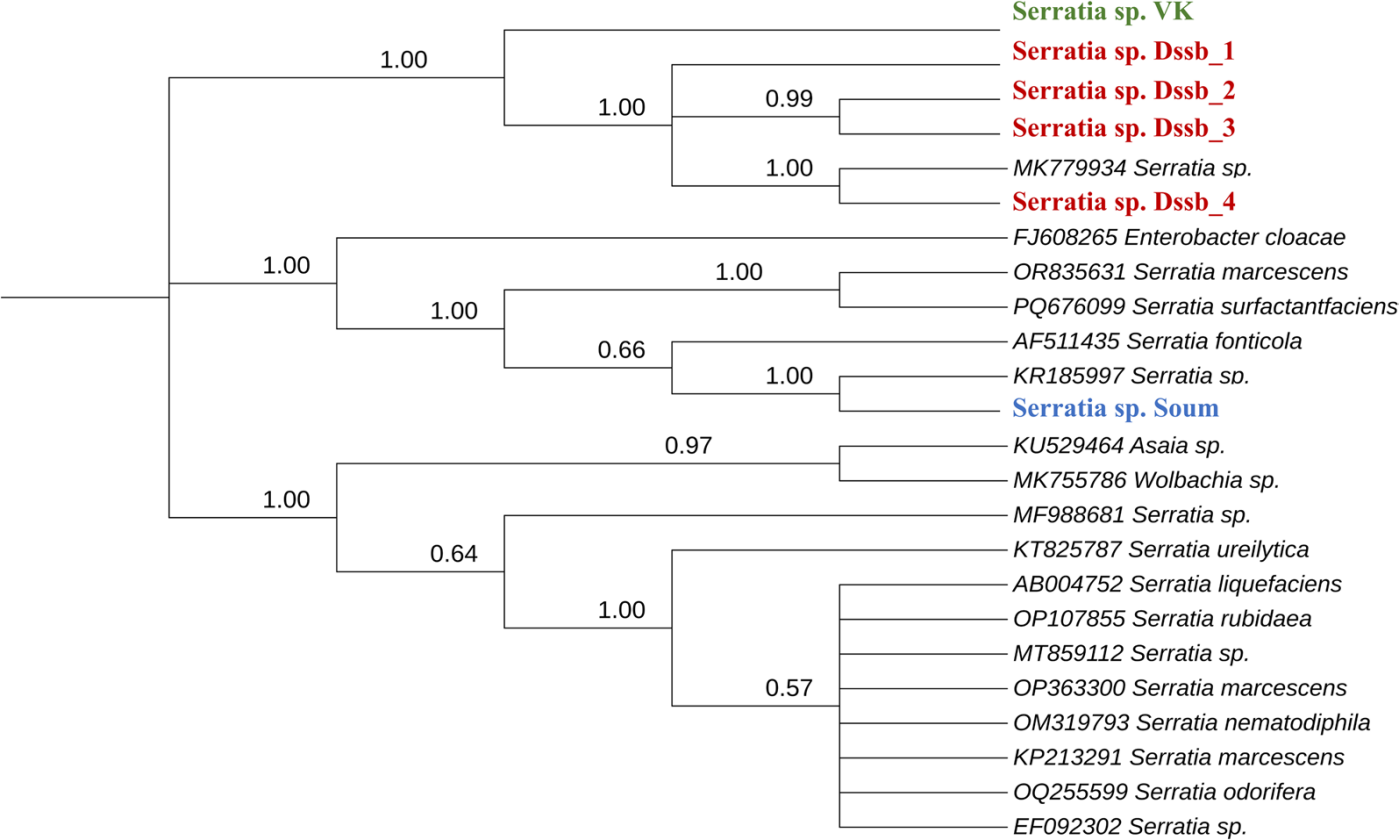
Evolutionary analysis of bacteria of the genus *Serratia.* The *Serratia* strains highlighted in red are the strains from Dioulassoba in the present study, while the strains highlighted in green and blue are from the Vallée du Kou and Soumousso, respectively. The phylogeny was inferred using the maximum likelihood method and the Kimura two-parameter model of nucleotide substitutions; the tree with the highest log likelihood (− 7356.08) is shown. The percentage of replicate trees in which the associated taxa clustered together (1000 replicates) is shown next to the branches [35]. The initial tree for the heuristic search was selected by choosing the tree with the superior log-likelihood between a neighbor-joining (NJ) tree [36] and a maximum parsimony (MP) tree. The NJ tree was generated using a matrix of pairwise distances computed using the p-distance [37]. The MP tree had the shortest length among 10 MP tree searches, each performed with a randomly generated starting tree. The rate model allowed for 13.42% of sites to be evolutionarily invariable (*I*). The analytical procedure encompassed 24 coding nucleotide sequences using first, second, third and non-coding positions with 1744 positions in the final dataset. Evolutionary analyses were conducted in MEGA12 [38] utilizing up to 7 parallel computing threads

**Fig. 7:**
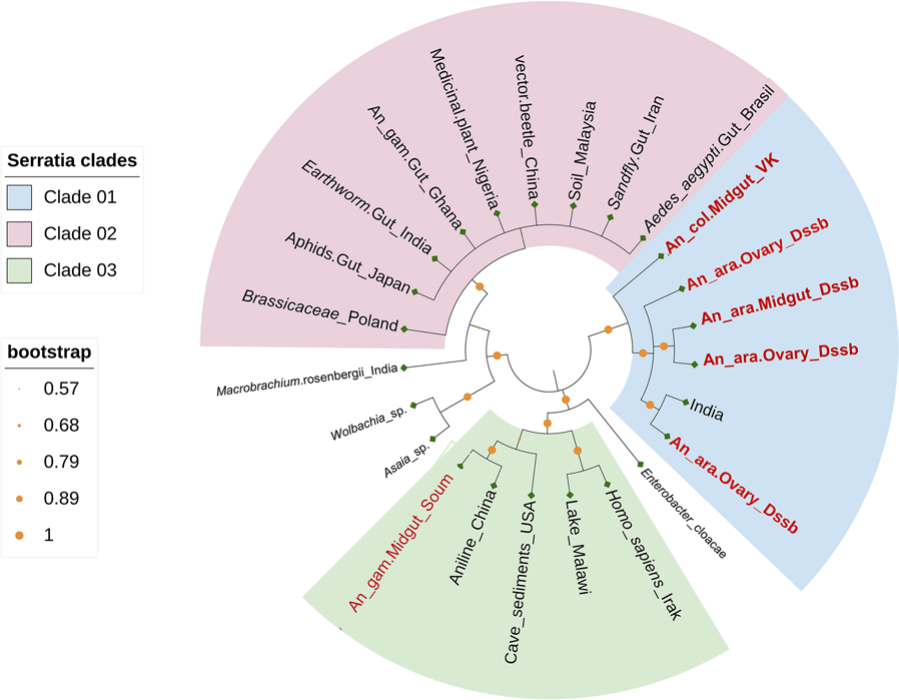
Phylogeny tree according sample source isolation. *Serratia* spp*.* samples collected in this study are labeled in red. An_ara, *Anopheles arabiensis*; An_col, *Anopheles coluzzii*; An_gam, *An_gambiae*

A phylogenetic relationship was established between the six samples identified in the present study, 17 Serratia strains and three additional bacterial taxa from the genera *Enterobacter, Wolbachia* and *Asaia* available in the NCBI database (Fig. 6). Sequences submitted to GenBank (Table 3) showed similarity levels ranging from 95.6% to 98.5% with *Serratia* sequences available in the database (Additional file 4: Table S1). *Serratia sp._Dssb1* and *Serratia sp._VK* (*Anopheles*’s_midgut, VK) were identified as the genus *Serratia,* with the percentage of identity being 97.2% and 95.6% respectively. However, our sample *Serratia* sp*._Dssb2* was 98.3% similar to *S. marcescens*, *Serratia* sp. _Dssb3 was 98.4% similar to *Serratia liquefaciens* and *Serratia* sp.*_Dssb4* and *Serratia* sp*._Soum* were 98.5% and 98.1%, respectively, similar to strain *S. ureilytica*. Phylogenetic analysis showed that the strains cluster with reference* Serratia* species, confirming their taxonomic assignment (Fig. 6). Moreover, the same sequences grouped according to their mosquito organ and collection site, highlighting that all our strains are grouped in the same clade, unlike *Serratia sp. Soum* isolated in the midgut of *An. gambiae* in Soumousso (Fig. 7).

**Table 3:** Information on the *Serratia* strains: sample ID, their collection sites and the accession numbers of the 16S ribosomal RNA gene sequence of* Serratia*

Samples ID	Collection site	GenBank ID
*Serratia sp._Dssb1*	Dioulassoba	PX438063
*Serratia sp*._Dssb2	Dioulassoba	PX438064
*Serratia sp*. _Dssb3	Dioulassoba	PX438065
*Serratia sp*._VK	Vallée du Kou	PX438066
*Serratia sp.* _Dssb4	Dioulassoba	PX438067
*Serratia sp._*Soum	Soumousso	PX438068

## Discussion

Previously reported results on the ability of *Serratia* spp*.* to block *Plasmodium* transmission suggest that *Serratia* could be used in the fight against malaria. However, very little is currently known about the distribution and biology of this bacterium in Burkina Faso, a country where malaria is endemic. In this study, we confirmed the presence of *Serratia* spp*.* in *Anopheles* populations in western Burkina Faso through bacterial 16S rRNA sequencing, with the results showing a prevalence of 13.3%. However, this prevalence is lower than that previously reported, particularly in Asia (16.2%, 51.4% and 21.1% in the Chinese provinces of Yunnan, Liaoning and Jiangsu, respectively) and in the West African sub-regions of Ghana (57% of the 14 isolates) and Côte d’Ivoire (75.5% of the microbiota in sensitive *An. coluzzii* populations) [12, 25, 26].

In our study, the prevalence of *Serratia* varied significantly according to *Anopheles* species. This significant variation between species of the *Anopheles gambiae* complex (*An. coluzzii, An. gambiae, An. arabiensis*) suggests that *Serratia* infection in different *Anopheles* species may be influenced by biological factors specific to each species. This result is not surprising because the *An. gambiae complex* shows significant genomic divergence [27], which could lead to different phenotypes, such as adaptation and persistence of colonization by microorganisms, between these species.

Our results showed that the prevalence of *Serratia* also varied according to the different collection sites. This significant variation in *Serratia* spp*.* highlights the importance of environmental factors in the composition of mosquito microbiota. Soumousso is a savannah area where cotton is cultivated, and the Vallée du Kou is a well-irrigated area where the main activity is rice cultivation with intensive use of pesticides, often in an unregulated way. Dioulassoba is an urban area where the level of sanitation is poor. Previous studies have shown that the use of pesticides is associated with mosquito resistance to insecticides [28, 29] and a reduction in the overall diversity and abundance of the microbiota [26].

We found that *Serratia* was present in both mosquito larvae and adults, but with a low prevalence in larvae. The presence of *Serratia* in *Anopheles* larvae, although at low levels, is an important observation because this bacterium can persist throughout metamorphosis until the adult mosquito stage, suggesting a potential transstadial transmission pathway, as reported in a previous study [30]. Such persistence of midgut bacteria across developmental transitions has been previously described in mosquitoes, where mechanisms involving meconial peritrophic membranes were proposed to explain the survival of certain bacteria during metamorphosis [31]. While general mechanisms for midgut bacterial persistence have been described, their applicability to *Serratia* remains unclear, highlighting the need for further investigation.

One of the main achievements of this study was the isolation of *Serratia* from various female organs, including the salivary gland, midgut, ovary, spermatheca and male testes. The presence of *Serratia* spp*.* in the midgut and salivary gland shows that this bacterium could possibly interact with *Plasmodium* during its developmental cycle, particularly at the oocyst stage, which occurs in the midgut, and on the sporozoites, which are located in the salivary gland. *Serratia* spp*.* has also been found in the male and female reproductive organs, showing the possibility of horizontal and vertical transmission under natural conditions, as previously reported in an experimental study [12, 30]. Among the different physiological states of the females, *Serratia* was significantly more frequent in gravid females than in unfed or recently blood-fed females. In fact, 24 h after a blood meal, the bacterial load increases exponentially, with only the most competent bacteria persisting and multiplying in this hostile atmosphere [32, 33]. Taken together, we conclude that *Serratia* has the ability to persist within female mosquitoes, which is a very important property for the use of symbiotic bacteria as bioagents.

Co-infection between *Plasmodium* and *Serratia* was found to be very low, with only one *Plasmodium*-positive mosquito also being positive for *Serratia*. This low prevalence of *P. falciparum*, however, may be partially attributable to the timing of the collection period (early dry season). This observation is in line with experimental data suggesting that certain *Serratia* strains can interfere with *Plasmodium* development by producing enzymes or activating immune responses [9, 12, 14, 30]..

The 16S rRNA gene analysis of our samples revealed high similarity with other previously described strains of the *Serratia* genus from several sources (soil, plant, other insects), confirming bacterial identity. Our sample *Serratia sp._Dssb*2 (*Anopheles*’s_ovary, Dioulassoba) was 98.6% similar to *S. marcescens* isolated from soil in Malaysia. Moreover, sample *Serratia sp.*_Dssb3 (*Anopheles*’s_midgut, Dioulassoba) was 98.4% similar to *S. liquefaciens* isolated from the gut of aphids in Japan. Also, our sample *Serratia_Dssb4 *(*Anopheles*’s_ovary, Dioulasso) and *Serratia sp._Soum* (*Anopheles*’s_midgut, Soumousso) were 98.5% and 98.1% respectively, similar to *S. ureilytica* isolated from a plant in Poland. However, our *Serratia sp._Dssb1* (*Anopheles*’s_ovary, Dioulasoba) and *Serratia sp._VK* (*Anopheles*’s_midgut, VK) sample were identified as the genus *Serratia* spp*.* with a percentage of identity of 97.2% and 95.6% respectively. Our results showed moderate diversity among our isolates but a diversity different from those reported in neighboring countries such as Ghana and Nigeria in *Anopheles* and in a medical plant [25, 34].

The mosquito collections were restricted to a specific method, specific period of time and three specific collection areas, all of which may have introduced biases. In addition, the relatively low prevalence of *Plasmodium* infections observed in our samples reduced the statistical power to detect associations with bacterial infections. Finally, while our results are preliminary, they highlight the need for further investigations, including the isolation of naturally occurring *Serratia* strains and experimental infections, to better assess the potential impact of this symbiont on *Plasmodium* transmission.

## Conclusions

Bacteria symbionts associated with mosquitoes are being intensively researched for their anti-parasitic and/or vector population suppression properties for new control malaria methods**.** This study is the first report on the molecular characterization of *Serratia* from different organs of the *An. gambiae* complex in Burkina Faso. However, to obtain a better understanding of the functional impact of these local strains and their use for controlling malaria transmission, additional higher resolution analyses such as whole genome sequencing, transcriptomic or proteomic analyses and the potential impact of this bacterium on malaria transmission in laboratory and semi-field experiment are recommended.

## Supplementary Information

**Additional file 1.Text S1.** Kerri Coon proposal.** Text S2.** Protocol for DNA extraction using 2% CTAB according to Myriam and Cécile (2003).

**Supplementary Material 2: Figure S1.** Agarose gel electrophoresis of the SINE 200X amplicons and *Plasmodium falciparum*.** A** Agarose gel electrophoresis of the SINE 200X amplicons. L, DNA marker (100–1500) bp;* An coluzzii:*, 479 bp; *An gambiae*, 249 bp; *An arabiensis*, 223 bp.** B** Agarose gel electrophoresis of the* Csp* gene for *P. falciparum* amplicons. Fragment sizes 450 bp. L, DNA marker (100–1500 bp); lanes 1–11, *P. falciparum*; lane 12, positive control; lane 14, negative control.

**Additional file 3: Figure S1. **Agarose gel electrophoresis of the 16S rRNA gene amplicons and *Serratia* spp.* luxS* gene.** A** Agarose gel electrophoresis of the 16S rRNA gene amplicons. Fragment sizes 1400–1500 bp. L, DNA marker (1 kb); lanes 1–23, positive samples; lane 24, positive control; lane 25, negative control.** B** Agarose gel electrophoresis of the* LuxS* gene amplicons. Fragment sizes 102 bp. L, DNA marker (100–1500 bp); lane 1, positive control; lanes 1–9, *Serratia* spp; lane 11, negative control.

**Additional file 4: Table S1.** BLAST alignment results.

**Additional file 5: Table S1.** Sequences use phylogenetic analysis.** Text S1.** Fasta Sequences.

**Additional File 6: Text S1. **Result of sequence alignments using the Muscle program in MEGA 12.0 software

**Additional file 7: Text S1.** R code.

## Data Availability

The 16S rRNA gene sequences and data for all analyses in this article with R code are available as supplementary files.

## Abbreviations

CTAB: Cetyl trimethyl ammonium bromide
Dssb: Dioulassoba
rRNA: Ribosomal ribonucleic acid
Soum: Soumousso
VK: Vallée du Kou
