# Supplementary material for: Molecular characterization of potential Plasmodium-Blocking Serratia spp. bacteria in field-caught malaria mosquito in Burkina Faso
Source: Parasit Vectors. 2025 Dec 21;19:47. doi: 10.1186/s13071-025-07191-2 (PMC12836870; doi:10.1186/s13071-025-07191-2)
Supplement: Supplementary file 1 — Additional file 1.Text S1. Kerri Coon proposal. Text S2. Protocol for DNA extraction using 2% CTAB according to Myriam and Cécile (2003). [file 13071_2025_7191_MOESM1_ESM.docx]

# Additional file 1 : Text S1. Protocol for DNA extraction using 2% CTAB, Myriam and Cécile,

1. Grind each mosquito in 200 μL of 2% CTAB ;

2. Place in a water bath at 65°C for 5 minutes ;

3. Add 200 μL of chloroform – Mix by inversion ;

4. Centrifuge for 5 minutes at 12,000 rpm at room temperature ;

5. Remove the upper phase and place it in another tube ;

6. Add 200 μL of isopropanol to this supernatant – Mix well by inversion ;

7. Centrifuge for 15 minutes at 12,000 rpm at room temperature.

8. Discard the isopropanol, drain thoroughly and add 200 μL of 70% ethanol ;

9. Centrifuge for 5 minutes at 12,000 rpm at room temperature ;

10. Discard the ethanol and leave for at least 20 minutes at room temperature ;

11. Dry the pellet for approximately 10 minutes in a speed-vac ;

12. Resuspend in 10 μL of H2O – do not vortex – Leave to stand on the bench overnight (or for half a day).

13. Store the DNA at -20°C and use 1 μL (pure) or 2 μL (diluted) of DNA for PCR analyses.
