# Supplementary figures and images for: Molecular characterization of potential Plasmodium-Blocking Serratia spp. bacteria in field-caught malaria mosquito in Burkina Faso
Source: Parasit Vectors. 2025 Dec 21;19:47. doi: 10.1186/s13071-025-07191-2 (PMC12836870; doi:10.1186/s13071-025-07191-2)

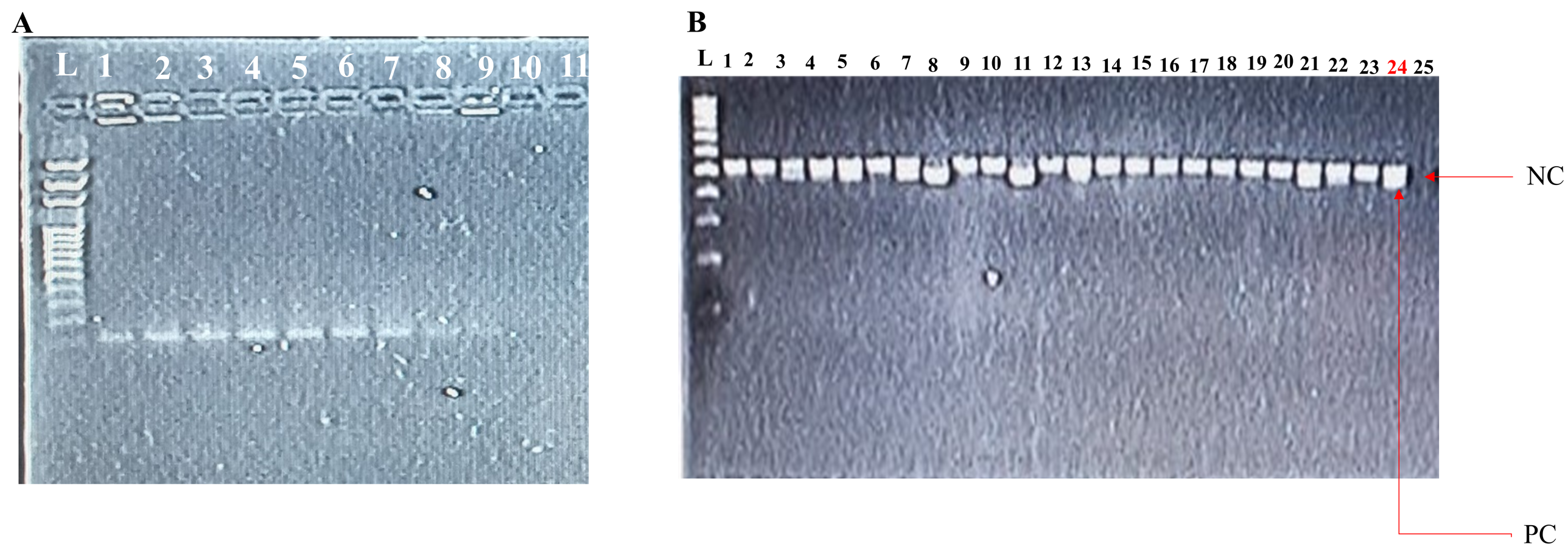

Supplement: Supplementary file 2 — Supplementary Material 2: Figure S1. Agarose gel electrophoresis of the SINE 200X amplicons and Plasmodium falciparum. A Agarose gel electrophoresis of the SINE 200X amplicons. L, DNA marker (100–1500) bp; An coluzzii:, 479 bp; An gambiae, 249 bp; An arabiensis, 223 bp. B Agarose gel electrophoresis of the Csp gene for P. falciparum amplicons. Fragment sizes 450 bp. L, DNA marker (100–1500 bp); lanes 1–11, P. falciparum; lane 12, positive control; lane 14, negative control. [file 13071_2025_7191_MOESM2_ESM.tif]
