# Supplementary material for: Molecular characterization of potential Plasmodium-Blocking Serratia spp. bacteria in field-caught malaria mosquito in Burkina Faso
Source: Parasit Vectors. 2025 Dec 21;19:47. doi: 10.1186/s13071-025-07191-2 (PMC12836870; doi:10.1186/s13071-025-07191-2)
Supplement: Supplementary file 4 — Additional file 4: Table S1. BLAST alignment results. [file 13071_2025_7191_MOESM4_ESM.docx]

# Additional file 4 : Table 1. Blast alignement result

| **Accession** | **Organism** | **% Identical Sites** | **E Value** | **Query coverage %** |
| --- | --- | --- | --- | --- |
|  | ***Serratia*_sp._(Anopheles's_ovary_Dioulasoba)01** |  |  |  |
| MK779934 | *Serratia* sp. (in: enterobacteria) strain DMS_D01 16S ribosomal RNA gene, partial sequence | 97.20 | 0 | 100 |
|  | ***Serratia*_sp._(Anopheles's_ovary_Dioulasoba)02** |  |  |  |
| MK779934 | *Serratia* sp. (in: enterobacteria) strain DMS_D01 16S ribosomal RNA gene, partial sequence | 98.41 | 0 | 100 |
| KP213291 | *Serratia* marcescens strain AQ07 16S ribosomal RNA gene, partial sequence | 98.27 | 0 | 100 |
| AB004752 | *Serratia* liquefaciens gene for 16S ribosomal RNA, partial sequence | 98.27 | 0 | 100 |
| [OM319793](https://sky-blast.com/accession/OM319793) | *Serratia* nematodiphila strain ZJPC33 16S ribosomal RNA gene, partial sequence | 98.12 | 0 | 100 |
| OQ255599 | *Serratia* odorifera strain S543 16S ribosomal RNA gene, partial sequence | 98.13 | 0 | 100 |
| OP107855 | *Serratia* rubidaea strain Os7 16S ribosomal RNA gene, partial sequence |  | 0 | 100 |
|  | ***Serratia*_liquefaciens_(Anopheles's_Stomach_Dioulassoba)04** |  |  |  |
| [AB004752](https://sky-blast.com/accession/AB004752) | *Serratia* liquefaciens gene for 16S ribosomal RNA, partial sequence | 98.43% | 0 | 100 |
|  | ***Serratia*_sp. 01_(Anopheles's_Stomach_Vallee_du_Kou)** |  |  |  |
| MF988681 | Uncultured *Serratia* sp. clone S 16S ribosomal RNA gene, partial sequence | 95.58% | 0 | 100 |
|  | ***Serratia*_ureilytica_(Anpheles's_ovary_Dioulasso)05** |  |  |  |
| KT825787 | *Serratia* ureilytica strain 24Kp1 16S ribosomal RNA gene, partial sequence | 98.47 | 0 | 100 |
|  | **S.ureilytica(An_stomach_Soum** |  |  |  |
| KR185997 | *Serratia* sp. Q11 16S ribosomal RNA gene, partial sequence | 99.71 | 0 | 100 |
| KT825787 | *Serratia* ureilytica strain 24Kp1 16S ribosomal RNA gene, partial sequence | 98.12 | 0 | 100 |
| AF511435 | *Serratia* fonticola 16S ribosomal RNA gene, partial sequence. | 97.11 | 0 | 100 |
