# Supplementary material for: Molecular characterization of potential Plasmodium-Blocking Serratia spp. bacteria in field-caught malaria mosquito in Burkina Faso
Source: Parasit Vectors. 2025 Dec 21;19:47. doi: 10.1186/s13071-025-07191-2 (PMC12836870; doi:10.1186/s13071-025-07191-2)
Supplement: Supplementary file 5 — Additional file 5: Table S1. Sequences use phylogenetic analysis. Text S1. Fasta Sequences. [file 13071_2025_7191_MOESM5_ESM.docx]

**Additional file 5 : Table 2. Sequences use phylogenetic analyse**

| Accession | **Others sequences use phylogenetic analyse** |
| --- | --- |
| PQ676099 | *Serratia* surfactantfaciens strain LOGO27 16S ribosomal RNA gene, partial sequence |
| MK755786 | *Wolbachia_sp.* |
| KU529464 | *Asaia_sp.* |
| FJ608265 | *Enterobacter cloacae* strain F34 16S ribosomal RNA gene, partial sequence |
| OP363300 | *Serratia* marcescens strain XAFb12 16S ribosomal RNA gene, partial sequence |
| OR835631 | *Serratia* marcescens strain JAQVM3 16S ribosomal RNA gene, partial sequence |
| MT859112 | *Serratia* sp. (in: enterobacteria) strain Tu2bii 16S ribosomal RNA gene, partial sequence |
| EF092302 | *Serratia* sp. P13 16S ribosomal RNA gene, partial sequence |
