# Supplementary material for: Molecular characterization of potential Plasmodium-Blocking Serratia spp. bacteria in field-caught malaria mosquito in Burkina Faso
Source: Parasit Vectors. 2025 Dec 21;19:47. doi: 10.1186/s13071-025-07191-2 (PMC12836870; doi:10.1186/s13071-025-07191-2)
Supplement: Supplementary file 7 — Additional file 7: Text S1. R code. [file 13071_2025_7191_MOESM7_ESM.docx]

**Additional file 7: Text S2. R code**

**######Base1**

#"Prevalence Serration according locality and anopheles species

library(dplyr)

library(tidyr)

library(Hmisc)

library(ggplot2)

data <- read.table("C:/Users/hp/Desktop/my thesis/Serratia spp_JAMOVI/Base_Haoua/Base_PrevSerratia_Pf.txt", header = TRUE, stringsAsFactors =TRUE)

summary(data)

View(data)

data<-data%>%drop_na(An_species)

data<-data%>%drop_na(Sex)

attach(data)

summary(data)

t1<-table(Serratia_Spp,Locality,An_species)

t2<-data.frame(t1,c(2,3))

t2

library(reshape2)

prev=dcast(t2, An_species+Locality~Serratia_Spp,value.var="Freq")

names(prev)[3] <- "uninf"

names(prev)[4] <- "inf"

prev$tot<-prev$uninf+prev$inf

library(Hmisc)

CI1<-binconf(prev$inf,prev$tot,method="wilson")

dh2<-cbind(prev,CI1)

dh2

library(ggplot2)

none <- element_blank()

dh2$Locality<-factor(dh2$Locality,levels = c("Dioulassoba","Soumousso","VK"),labels = c("D","S","VK"))##Changer le nom pour reduire

figA<-ggplot(dh2,aes(x=Locality,y=PointEst, fill= Locality))+

geom_bar(stat="identity",color="black")+

geom_errorbar(aes(ymin=Lower, ymax=Upper), color="black",width=.2,size=0.5)+

facet_wrap(~An_species , nrow = 1) +

xlab("\n Locality")+ ylab("Serratia spp_prevalence\n")+

coord_cartesian(ylim = c(0, 1)) + scale_y_continuous(breaks=seq(0, 1, 0.25))+

theme(legend.position = "none")+

theme(axis.text=element_text(size= 12,colour="black"),axis.title=element_text(size=12))+

theme(axis.line = element_line(colour = "black"))+

theme(strip.text.x = element_text(size = 12, face = 'bold.italic'))+

theme(axis.line = element_line(colour = "black"))+

theme(legend.background = element_rect(fill="white"),legend.key = element_rect(fill = "white", color = NA))+

theme(panel.grid.major = none,panel.grid.minor = none)+

theme(panel.background = none) + theme(panel.border = none)+scale_fill_manual(values=c("darkred","olivedrab","royalblue"),labels=expression("D","S","VK"))

figA

###########Prevalence en pourcenetage et renommée locality

figA <- ggplot(dh2, aes(x = Locality, y = PointEst * 100, fill = Locality)) + geom_bar(stat = "identity", color = "black") + geom_errorbar(aes(ymin = Lower * 100, ymax = Upper * 100),color = "black", width = .2, size = 0.5) +facet_wrap(~An_species, nrow = 1) + xlab("\n Locality") +ylab("Serratia spp. prevalence (%)\n") +coord_cartesian(ylim = c(0, 75)) + scale_y_continuous(breaks = seq(0, 75, 25)) +theme_bw() + theme(legend.position = "none",

axis.text.x = element_text(size = 12, colour = "black"), axis.text.y = element_text(size = 12, colour = "black"), axis.title = element_text(size = 12), axis.line = element_line(colour = "black"), strip.text.x = element_text(size = 12, face = 'bold.italic'), strip.background = element_rect(fill = "gray90", color = "black"), panel.grid.major = element_blank(), panel.grid.minor = element_blank(), panel.border = element_blank(), panel.background = element_blank(), plot.background = element_rect(fill = "white", color = NA))

figA1

####Statistique

###### prevalence

mod1<-glm(Serratia_Spp~Locality*An_species,family=binomial)

summary(mod1)

mod2<-glm(Serratia_Spp~Locality+An_species,family=binomial)

summary(mod2)

anova(mod1,mod2,test="Chi")

anova(mod1,mod2,test="Chi")

mod3<-glm(Serratia_Spp~Locality,family=binomial)

mod4<-glm(Serratia_Spp~An_species,family=binomial)

anova(mod1,mod4,test="Chi")

###prevalence en fonction du statut

data<-data%>%drop_na(Status)

summary(data)

attach(data)

h1<-table(Serratia_Spp, Status)

h2<-data.frame(h1,c(2,3))

h2

library(reshape2)

prev=dcast(h2, Status~Serratia_Spp,value.var="Freq")

names(prev)[2] <- "uninf"

names(prev)[3] <- "inf"

prev$tot<-prev$uninf+prev$inf

library(Hmisc)

CI<-binconf(prev$inf,prev$tot,method="wilson")

dh<-cbind(prev,CI)

dh

library(ggplot2)

none <- element_blank()

figB<-ggplot(dh,aes(x=Status,y=PointEst, fill= Status))+

geom_bar(stat="identity",color="black",width=0.6)+

geom_errorbar(aes(ymin=Lower, ymax=Upper), color="black",width=0.2,size=0.5)+

xlab("\n Status")+ ylab("Serratia spp_prevalence\n")+

coord_cartesian(ylim = c(0, 0.5)) + scale_y_continuous(breaks=seq(0, 1, 0.25))+

theme(legend.position = "none")+

theme(axis.text=element_text(size= 12,colour="black"),axis.title=element_text(size=12))+

theme(axis.line = element_line(colour = "black"))+

theme(strip.text.x = element_text(size = 12, face = 'bold.italic'))+

theme(axis.line = element_line(colour = "black"))+

theme(legend.background = element_rect(fill="white"),legend.key = element_rect(fill = "white", color = NA))+

theme(panel.grid.major = none,panel.grid.minor = none)+

theme(panel.background = none) + theme(panel.border = none)+scale_fill_manual(values=c("maroon","Goldenrod","darkgreen"),labels=expression("blood_fed","Gravid","Jeun"))

figB

#########"FigB prevalence status in %

figB <- ggplot(dh, aes(x = Status, y = PointEst * 100, fill = Status)) +

geom_bar(stat = "identity", color = "black", width = 0.5) +

geom_errorbar(aes(ymin = Lower * 100, ymax = Upper * 100), color = "black", width = 0.2, size = 0.5) +

xlab("\n Status") +

ylab("Serratia spp prevalence (%)\n") +

coord_cartesian(ylim = c(0, 50)) +

scale_y_continuous(breaks = seq(0, 100, 25)) +

theme(legend.position = "none",

axis.text = element_text(size = 12, colour = "black"),

axis.title = element_text(size = 12),

axis.line = element_line(colour = "black"),

strip.text.x = element_text(size = 12, face = 'bold.italic'),

legend.background = element_rect(fill = "white"),

legend.key = element_rect(fill = "white", color = NA),

panel.grid.major = element_blank(),

panel.grid.minor = element_blank(),

panel.background = element_blank(),

panel.border = element_blank())

figB

chisq.test(h1)

####### **Base 2**

#**###** prevalence Serratia according of female organs

###prevalence en fonction des organes

base <- read.table("C:/Users/hp/Desktop/my thesis/Serratia spp_JAMOVI/Base_Haoua/Prev-Serratia_Organs.txt", header = TRUE, stringsAsFactors = TRUE)

###prevalence en fonction du statut

summary(base)

attach(base)

attach(base)

h1<-table(Serratia, Organes )

h2<-data.frame(h1,c(2,3))

h2

library(reshape2)

prev=dcast(h2,Organes~Serratia,value.var="Freq")

names(prev)[2] <- "uninf"

names(prev)[3] <- "inf"

prev$tot<-prev$uninf+prev$inf

library(Hmisc)

CI<-binconf(prev$inf,prev$tot,method="wilson")

dh<-cbind(prev,CI)

dh

library(ggplot2)

none <- element_blank()

figC<-ggplot(dh,aes(x=Organes,y=PointEst, fill= Organes))+

geom_bar(stat="identity",color="black",width=0.6)+

geom_errorbar(aes(ymin=Lower, ymax=Upper), color="black",width=0.2,size=0.5)+

xlab("\n Organes")+ ylab("Serratia_prevalence\n")+

coord_cartesian(ylim = c(0, 1)) + scale_y_continuous(breaks=seq(0, 1, 0.25))+

theme(legend.position = "none")+

theme(axis.text=element_text(size= 12,colour="black"),axis.title=element_text(size=12))+

theme(axis.line = element_line(colour = "black"))+

theme(strip.text.x = element_text(size = 12, face = 'bold.italic'))+

theme(axis.line = element_line(colour = "black"))+

theme(legend.background = element_rect(fill="white"),legend.key = element_rect(fill = "white", color = NA))+

theme_classic()+

theme(panel.background = none) + theme(panel.border = none)+scale_fill_manual(values=c("darkred","olivedrab","royalblue","gray70"),labels=expression("Ovary","Spermathca","Stomach","Glande_saliva"))

figC

chisq.test(h1)

######Proportion en pourcentage

FigC1 <- ggplot(dh, aes(x = Organes, y = PointEst * 100, fill = Organes)) +

geom_bar(stat = "identity", color = "black", width = 0.5) +

geom_errorbar(aes(ymin = Lower * 100, ymax = Upper * 100), color = "black", width = 0.2, size = 0.5) +

xlab("\n Organes") +

ylab("Serratia_prevalence (%)\n") +

coord_cartesian(ylim = c(0, 100)) +

scale_y_continuous(breaks = seq(0, 100, 25)) +

theme_classic() + # fond blanc, sans fond gris

theme(

legend.position = "none", # enlève la légende

axis.text = element_text(size = 12, colour = "black"),

axis.title = element_text(size = 12),

axis.line = element_line(colour = "black"),

strip.text.x = element_text(size = 12, face = 'bold.italic'),

plot.background = element_rect(fill = "white", color = NA)

)

FigC1

#########**Base 3**

###prevalence Pf

data <- read.table("C:/Users/hp/Desktop/my thesis/Serratia spp_JAMOVI/Base_Haoua/Base_PrevSerratia_Pf.txt", header = TRUE, stringsAsFactors =TRUE)

library(ggplot2)

library(dplyr)

data <- data %>% drop_na(Pf)

data<-data%>%drop_na(Pf)

summary(data)

attach(data)

T1<-table(Serratia_Spp, Pf)

T2<-data.frame(h1,c(2,3))

T2

library(reshape2)

prev=dcast(T2, Pf~Serratia_Spp,value.var="Freq")

names(prev)[2] <- "uninf"

names(prev)[3] <- "inf"

prev$tot<-prev$uninf+prev$inf

library(Hmisc)

CT<-binconf(prev$inf,prev$tot,method="wilson")

Th<-cbind(prev,CT)

Th

# Conversion des variables en facteurs avec des labels clairs

data <- data %>%

mutate( Serratia_Label = factor(Serratia_Spp, levels = c(0, 1), labels = c("Serratia -", "Serratia +")), Pf_Label = factor(Pf, levels = c(0, 1), labels = c("Pf -", "Pf +")) )

summary_data <- data %>%

group_by(Serratia_Label, Pf_Label) %>%

summarise(n = n(), .groups = "drop") %>%

group_by(Serratia_Label) %>%

mutate(freq = n / sum(n))

# Diagramme empilé à 100 % avec couleurs spécifiques

fig4 <- ggplot(summary_data, aes(x = Serratia_Label, y = freq, fill = Pf_Label)) +

geom_col(width = 0.6, color = "white") +

# Couleurs manuelles respectant Pf+ = marron, Pf- = bleu ciel

scale_fill_manual(values = c("Pf +" = "maroon", "Pf -" = "skyblue"), breaks = c("Pf +", "Pf -"),

# ordre dans la légende

labels = c("Pf +", "Pf -")

) +

geom_text(

aes(label = scales::percent(freq, accuracy = 0.1)),

position = position_stack(vjust = 0.5),

size = 4,

color = "white"

) +

scale_y_continuous(labels = scales::percent_format()) +

labs(

title = "Prévalence de Pf selon la présence de Serratia (barres 100 %)",

x = "Présence de Serratia",

y = "Proportion (%)",

fill = "Statut Pf"

) +

theme_minimal() +

theme(

panel.background = element_blank(),

panel.border = element_blank() )

fig4

chisq.test(T1)
